# Genome Wide Identification, Evolutionary, and Expression Analysis of VQ Genes from Two *Pyrus* Species

**DOI:** 10.3390/genes9040224

**Published:** 2018-04-23

**Authors:** Yunpeng Cao, Dandan Meng, Muhammad Abdullah, Qing Jin, Yi Lin, Yongping Cai

**Affiliations:** School of Life Sciences, Anhui Agricultural University, Hefei 230036, China; xfcypeng@126.com (Y.C.); mdd5749@163.com (D.M.); abdullahpadana@hotmail.com (M.A.); qingjin@ahau.edu.cn (Q.J.); linyi320722@163.com (Y.L.)

**Keywords:** VQ, orthologous, *P. bretschneideri*, *P. communis*, expression

## Abstract

The VQ motif-containing gene, a member of the plant-specific genes, is involved in the plant developmental process and various stress responses. The VQ motif-containing gene family has been studied in several plants, such as rice (*Oryza sativa*), maize (*Zea mays*), and Arabidopsis (*Arabidopsis thaliana*). However, no systematic study has been performed in *Pyrus* species, which have important economic value. In our study, we identified 41 and 28 VQ motif-containing genes in *Pyrus bretschneideri* and *Pyrus communis*, respectively. Phylogenetic trees were calculated using *A. thaliana* and *O. sativa* VQ motif-containing genes as a template, allowing us to categorize these genes into nine subfamilies. Thirty-two and eight paralogous of VQ motif-containing genes were found in *P. bretschneideri* and *P. communis*, respectively, showing that the VQ motif-containing genes had a more remarkable expansion in *P. bretschneideri* than in *P. communis*. A total of 31 orthologous pairs were identified from the *P. bretschneideri* and *P. communis* VQ motif-containing genes. Additionally, among the paralogs, we found that these duplication gene pairs probably derived from segmental duplication/whole-genome duplication (WGD) events in the genomes of *P. bretschneideri* and *P. communis*, respectively. The gene expression profiles in both *P. bretschneideri* and *P. communis* fruits suggested functional redundancy for some orthologous gene pairs derived from a common ancestry, and sub-functionalization or neo-functionalization for some of them. Our study provided the first systematic evolutionary analysis of the VQ motif-containing genes in *Pyrus*, and highlighted the diversification and duplication of VQ motif-containing genes in both *P. bretschneideri* and *P. communis*.

## 1. Introduction

The VQ motif-containing gene, a member of the plant-specific genes, is involved in the plant developmental process and various stress responses. The VQ motif-containing gene encoding domain contains a conserved VQ motif, which possesses 50–60 amino acids with a high conserved FXXhVQXhTG (h refers to hydrophobic amino acids and x refers to any amino acids) [1]. The VQ domain plays a critical role in the biological function of VQ motif-containing proteins. For example, the mutation of *AtVQ14* (i.e., changes from IVQQ to EDLE) in the VQ domain leads to the smaller seed of the mutant strain, while mutations in other locations do not have this phenotype [2]. It is reported that the first VQ motif-containing protein was identified from *Arabidopsis thaliana* using the yeast two-hybrid method [1]. Subsequently, the VQ motif-containing genes have been identified in many plants. A total of 39, 18, 74, 18, 61, 34, and 23 members have been identified in rice, moss, soybean, grape, maize, *A. thaliana*, and *Fragaria vesca*, respectively [3,4,5,6,7,8]. Evolutionary analysis shows that members of the VQ motif-containing gene family can be clustered into 9 groups, named from I-X, and further reveals the molecular mechanisms of these genes [3,9]. Among them, some closely related VQ motif-containing genes, which belong to the same subgroup, may be functionally redundant, such as *AtVQ1* and *AtVQ10* (group VIII), *AtVQ24* and *AtVQ15* (group X), and *AtVQ12* and *AtVQ29* (group IV) [3].

The published articles have reported that VQ motif-containing proteins participate not only in plant stress response to drought; salt; and pathogenic bacteria, fungi, and oomycetes, but also in the regulation of various life processes of plants, such as the development of seeds, hypocotyls, flowers, leaves, and fruits [3,4,5,6,7,9,10,11,12]. Remarkably, some VQ motif-containing proteins may form complexes with WRKYs through physical interactions [13], such as *AtVQ23* and *AtVQ16* that appear to act as activators of *AtWRKY33* in plant defenses [13]. AtVQ15 (AtCaMBP25) is a calmodulin-binding protein, and its overexpression transgenic plants are highly sensitive to osmotic stress and NaCl in seed germination and seedling growth [14]. Transgenic plants overexpressing *AtVQ21* (MKS1) showed significantly increased resistance to *Pseudomonas syringae* [15], while showing decreased resistance to *Botrytis cinerea* [16,17]. The *AtVQ29* plays an important role in the light morphogenesis of seedlings in *A. thaliana* [10]. For example, the expression level of *AtVQ29* in stems is significantly higher than that in roots, rosette leaves, flowers, and pods [10]. Transgenic plants overexpressing *AtVQ29* led to significantly longer hypocotyl lengths than the wild type under far-red or low light conditions, while the *A. thaliana* mutant plants’ hypocotyl lengths were significantly shorter than that of wild type [10]. The mutant plants of *A. thaliana AtVQ8* gene showed yellowish-green leaves and growth retardation throughout their whole growth and development period, and the growth of overexpression plants of *VQ17*, *VQ18,* and *VQ22* was also severely inhibited [3].

Recently, some published papers have confirmed that there are differences in the evolutionary pattern of gene families between adjacent species [8,18]. Zhong et al. [8] explored the evolution and functional of VQ motif-containing genes in six species of *Fragaria*, and suggested that the expansion pattern of this gene family was different among species [8]. Both *Fragaria* and *Pyrus* belong to Rosaceae family. *Pyrus*, which produces fruits that have great commercial value, is also one of the most important economical crops worldwide. Compared to the largely well characterized VQ motif-containing genes in *A. thaliana* [9,19], VQ motif-containing genes have not been functionally characterized in *Pyrus* (i.e., *Pyrus bretschneideri* and *Pyrus communis*). It is well-known that *P. bretschneideri* and *P. communis* have undergone at least two rounds of whole-genome duplication (WGD) events [20,21], which play an essential role in the expansion of the genes. Previous studies have reported that orthologous genes may contribute to some of the evolutionary innovations and may develop non-function, sub-function, or neo-function after evolving from a common ancestry in plants [22,23]. Thus, it is of interest to characterize orthologous VQ motif-containing genes between *P. bretschneideri* and *P. communis*. Our study identified 41 and 28 VQ motif-containing genes in *P. bretschneideri* and *P. communis*, respectively, and comprehensively characterized their evolutionary outcomes and gene duplication types based on the microcollinearity, gene structure, and expression patterns analyses. 

## 2. Materials and Methods

### 2.1. Identification and Classification of P. bretschneideri and P. communis VQ Motif-Containing Genes

Predicted proteomes from *P. bretschneideri* (http://gigadb.org/site) and *P. communis* (https://www.rosaceae.org) were selected and downloaded. The VQ motif-containing genes of *A. thaliana* were obtained from The Arabidopsis Information Resource (TAIR) database (http://arabidopsis.org). According to the Hidden Markov Model (HMM) profile of VQ domains (PF05678) and *A. thaliana* VQ motif-containing proteins, we performed basic local alignment search tool (BLAST) (E-value = 1 × 10^−3^) searches using HMMER3 software [24]. These putative VQ motif-containing sequences, which were confirmed to contain the VQ domains by the National Center for Biotechnology Information (NCBI) Conserved Domain database, Pfam database, and SMART database [25,26,27], were used for further analysis. The VQ motif-containing sequences from *Pyrus* and *A. thaliana* were aligned using MAFFT (Multiple Alignment using Fast Fourier Transform) with default parameters [28]. According to the alignment results, we constructed a phylogenetic tree using the neighbor-joining (NJ) method, as implemented in MEGA 5 software [29]. The classification information of VQ motif-containing genes was extracted from the previously published articles [9]. 

### 2.2. Chromosome Locations and Microcollinearity Analysis of Pyrus VQ Motif-Containing Genes

In order to understand the chromosome location information, we obtained the GFF (General Feature Format) files of *P. bretschneideri* and *P. communis* from GigaDB (http://gigadb.org/site) and GDR database (https://www.rosaceae.org), respectively. Protein BLAST (BLASTP) search was carried out against *P. bretschneideri* and *P. communis* genomes, with an E-value below 1 × 10^5^. According to the results of BLASTP and GFF, we determined the genome-wide duplication (GWD)/segmental duplication using MCScanX software [30]. The Ka (non-synonymous substitution)/Ks (synonymous substitution), Ka, and Ks values were estimated using DnaSP 5.0 software [31]. The microcollinearity visualization of the duplicated VQ motif-containing genes was performed by our Perl script (Supplementary Data S1). The Circos software was used to map the chromosome location among the *P. bretschneideri* and *P. communis* genomes [32]. 

### 2.3. Intron–Exon Structure and Domain Search of P. bretschneideri and P. communis VQ Motif-Containing Genes

According to the extracted information from the GFF files, we diagrammed the domain and intron–exon structures of *P. bretschneideri* and *P. communis* using R software and our Perl script (Supplementary Data S1). The domain information of *P. bretschneideri* and *P. communis* VQ motif-containing genes was obtained from the Pfam database [27]. The multiple EM for motif elicitation (MEME) online tool was used to identify the conserved VQ motifs with the following parameters: optimum motif length = 6–200 residues; maximum number of motifs = 20; the number of repetition = any [33].

### 2.4. Expression Data Analysis

To determine the expression of *PbVQ* genes in *P. bretschneideri*, RNA-Seq data were obtained from the NCBI database. The accession numbers and sample details or treatments for these data are presented in the availability of data and materials section. The clean reads, which were removed low-quality base-calls (Q < 20) using FASTX-toolkit (http://hannonlab.cshl.edu/fastx_toolkit), were mapped to the reference genome using TopHat2 software with default parameters, and were assembled using Cufflinks software [34,35]. The expression profiles were visualized using R software with our R script (Supplementary Data S1). 

### 2.5. Expression Correlation of Homologous VQ Motif-Containing Genes between P. bretschneideri and P. communis

Expression profiles of homologous VQ motif-containing genes were manually gathered from RNA-Seq data. Using Pearson’s correlation coefficient, we calculated the similarity between the expression patterns of the homologous gene pair. According to previous studies [36,37], significant values were suggested to confirm the degree of expression diversity. Generally, *r* < 0.3, 0.3 < *r* < 0.5, and *r* > 0.5 indicates divergence, ongoing divergent, and non-divergence, respectively [36,37].

### 2.6. Plant Material, RNA Extraction, and Real-Time Polymerase Chain Reaction Analysis

The ‘Dangshansuli’ (*P. bretschneideri*) and ‘Starkrimson’ (*P. communis*) were picked from 30-year-old pear trees grown on a pear orchard (Dangshan, Anhui, China). In the present study, we selected ten healthy and robust trees, which were managed in a consistent manner. When the trees were at the bud stage (i.e., April 2016), we selected short branches bearing buds of similar developmental stages from the middle-crown area on the south side of each tree, and then marked them. In each short branch, only two fruits were kept. We collected the fruits from seven developmental stages, including fruit_stage1 (15 days after full blooming (DAB)), fruit_stage2 (30 DAB), fruit_stage3 (55 DAB), fruit_stage4 (85 DAB), fruit_stage5 (115 DAB), fruit_stage6 (mature stage), and fruit_stage7 (fruit senescence stage), respectively. After removing the peels of the fruits, we extracted total RNA of flesh for the synthesis of the first-strand complementary DNA (cDNA) using Trizol reagent (Invitrogen, Carlsbad, CA, USA). The quantitative real-time PCR (qRT-PCR) reactions were executed with 40 cycles in the Bio-rad CFX96 Real-Time PCR Detection system (BioRad, Hercules, CA, USA) using the TransStart Tip Green qPCR SuperMix (TransGen Biotech, Beijing, China). According to a previous workflow, the normalization of each reaction threshold cycle (Ct) value was determined by pear tubulin gene [38,39]. Beacon Designer v7.9 was used to design specific primers of the qRT-PCR analysis [18,39], which are presented in Table S1. All expression data were obtained from three biological repeats. The SPSS v19.0 software was used to perform statistical significance with the *t* test [18,39]. 

### 2.7 Availability of Data and Materials 

Stop-growth pollen tube, Accession: SRX1356346; Pollen tube, Accession: SRX1356343; Hydrated pollen grains, Accession: SRX1356152; Mature pollen grains of pear, Accession: SRX1356151; Pear fruit developmental stage 1 (15DAB), Accession: SRX1595645; Pear fruit developmental stage 2 (30DAB), Accession: SRX1595646; Pear fruit developmental stage 3 (55DAB), Accession: SRX1595647; Pear fruit developmental stage 4 (85 DAB), Accession: SRX1595648; Pear fruit developmental stage 5 (115 DAB), Accession: SRX1595650; Pear fruit developmental stage 6 (mature stage), Accession: SRX1595651; Pear fruit developmental stage 7 (fruit senescence stage), Accession: SRX1595652; Pear pericarp-russet, Accession: SRX707274; Pear pericarp-green, Accession: SRX316593; Pear leaf with salt treatment, Accession: SRX525736; Pear leaf with inoculated black spot *(Alternarlia alternate*) 2, Accession: SRX864865; Pear leaf with inoculated distilled water 1, Accession: SRX864860; Pear leaf with inoculated distilled water 2, Accession: SRX864862; Pear leaf with inoculated black spot (*Alternarlia alternate*) 3, Accession: SRX864861; Pear fruit with Gibberellin treatment, Accession: SRX532394.

## 3. Results

### 3.1. Identification and Classification of P. bretschneideri and P. communis VQ Motif-Containing Genes

**Figure 1:**
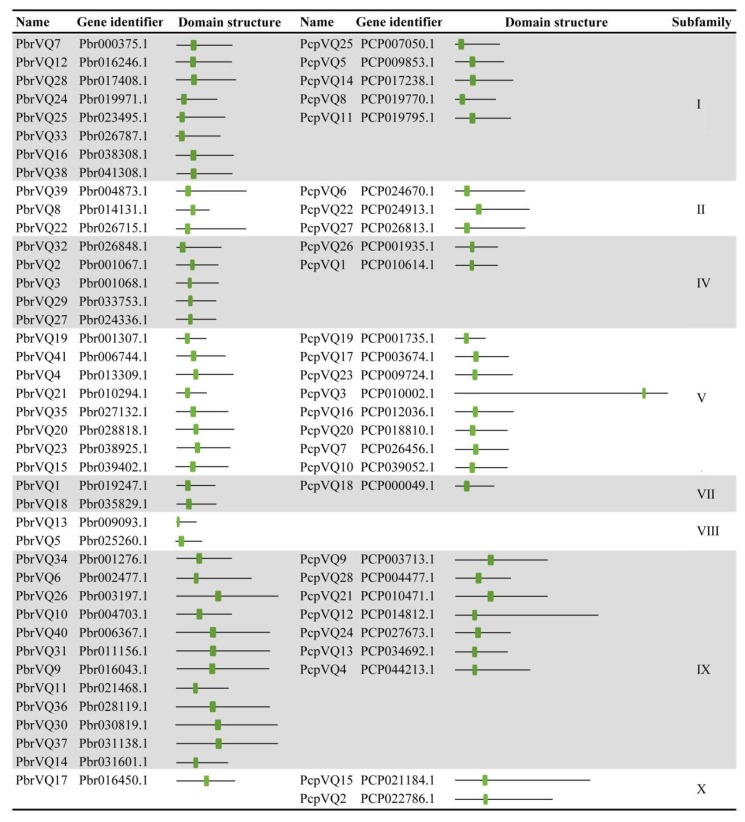
Summary of *Pyrus bretschneideri* and *Pyrus communis* VQ motif-containing genes. The classification information of VQ motif-containing genes was extracted from previously published articles [9]. The VQ domain is indicated in green.

To identify the VQ motif-containing gene family members encoded by *P. bretschneideri* and *P. communis* genomes, we searched for these genomes using HMMER 3.0 software and the Pfam database with the VQ domain (PF05678). We identified 41 and 28 non-redundant VQ motif-containing genes for the following analysis in *P. bretschneideri* and *P. communis*, respectively. Detailed information (including gene identifier, domain structure, and subfamily) concerning VQ motif-containing genes is listed in Figure 1.

**Figure 2:**
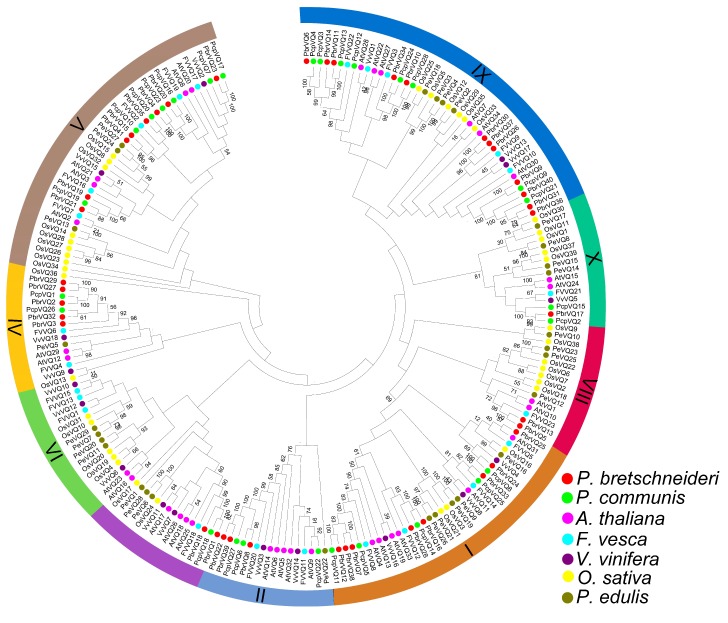
Classification and phylogenetic relationships of *P. bretschneideri* and *P. communis* VQ motif-containing genes. The neighbor-joining (NJ) tree was constructed by the full-length amino acid sequences using MEGA 5 software. According to the previously published articles [9], we divided these sequences into nine different subfamilies (I-X). Proteins from *P. bretschneideri*, *P. communis*, *Arabidopsis thaliana*, *Fragaria vesca*, *Vitis vinifera*, *Oryza sativa*, and *Phyllostachys edulis* are denoted by red, green, pink, blue, purple, yellow, and brown circles, respectively.

The classification of VQ motif-containing genes subfamilies has been carried out by Pascal Pecher et al. [13]. To further understand the phylogenetic relationships of VQ motif-containing gene family members in *P. bretschneideri* and *P. communis*, a NJ phylogenetic tree was constructed. As shown in Figure 2, the VQ proteins from monocotyledonous (*Oryza sativa* and *Phyllostachys edulis*) and dicotyledonous (*P. bretschneideri*, *P. communis*, *Vitis vinifera*, *A. thaliana,* and *F. vesca*) could be divided into nine subfamilies (I-X), based on the previous studies [9]. Remarkably, when compared with the other subfamilies, we noticed that the V and IX subfamily member sizes were significantly larger. These results were consistent with previous articles on *A. thaliana*, *O. sativa,* and *Z. mays* VQ motif-containing genes [4,7,19]. However, we also found that some subfamilies only contained VQ motif-containing genes from one or several species, indicating that they might have undergone dramatic changes after their origination during the long evolutionary period. For example, subfamily II contained *A. thaliana*, *P. edulis*, *F. vesca*, *P. communis*, *P. bretschneideri*, and *V. vinifera* VQ motif-containing genes, while subfamily VIII only contained *P. edulis*, *F. vesca*, *A. thaliana*, *O. sativa*, and *P. bretschneideri* VQ motif-containing genes. Interestingly, VQ motif-containing genes from *P. bretschneideri* and *P. communis* showed a higher similarity with each other, based on the evolutionary relationships. This was not surprising because *P. bretschneideri* and *P. communis* belong to *Pyrus* species. 

### 3.2. Chromosome Locations and Duplication Events of P. bretschneideri and P. communis VQ Motif-Containing Genes

**Figure 3:**
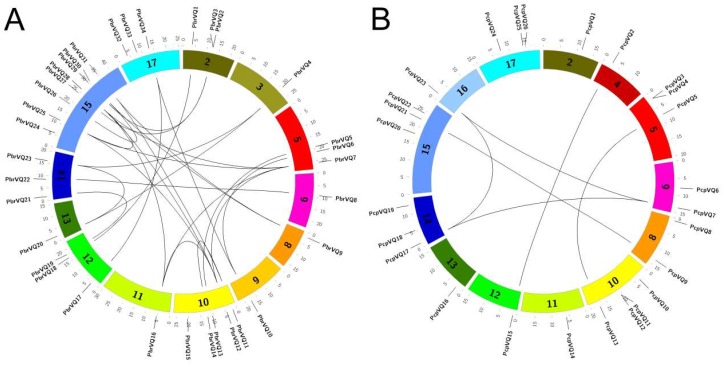
Chromosome location and duplication events analysis in both *P. bretschneideri* (**A**) and *P. communis* (**B**) VQ motif-containing genes. The outermost scale represents the megabases (Mb). The location of each VQ motif-containing genes is marked with a grey line using Circos software. The duplication gene pairs were linked by black lines. Remarkably, because *PbrVQ35*, *PbrVQ36*, *PbrVQ37*, *PbrVQ38*, *PbrVQ39*, *PbrVQ40*, *PbrVQ41*, *PcpVQ27,* and *PcpVQ28* were located on the scaffold, the location of these genes is not shown in Figure 3.

The *Pyrus* genome contained 17 chromosomes. To further understand the distribution of VQ motif-containing genes in the *Pyrus* genome, we mapped *P. bretschneideri* and *P. communis* VQ motif-containing genes to chromosome positions. In *P. bretschneideri*, the distribution of VQ motif-containing genes was among the chromosomes 2, 3, 5, 6, 8, 9, 10, 11, 12, 13, 14, 15, and 17, especially in chromosome 15 (Figure 3A). In *P. communis*, the distribution of VQ motif-containing genes was among the chromosomes 2, 4, 5, 6, 8, 10, 11, 12, 13, 14, 15, 16, and 17, especially in chromosome 10 (Figure 3B). As we know, the pear genome has undergone two WGD events. It has been reported that a recent WGD was estimated at 30–45 MYA (Millions of years ago) (Ks ∼ 0.15–0.3), while an ancient WGD was estimated at ∼140 MYA (Ks ∼ 1.5–1.8). To gain insight into the relationship between VQ motif-containing gene expansions and WGD events, we identified 32 and 8 collinearity events among *P. bretschneideri* and *P. communis*, respectively (Figure 3). In *P. bretschneideri*, we found that most Ks ratios of these collinearity events varied from 0.15–0.3 (Table S2). However, only three Ks ratios ranging from 0.15–0.3 were detected in *P. communis*. Remarkably, the ancient WGD events were not found in the VQ motif-containing genes of these two species, because their Ks values were not distributed in the 1.5–1.8 range (Table S2). The results indicated that a recent GWD might have contributed to the expansions of *P. bretschneideri* VQ motif-containing gene members. At the same time, these results further explain why the number of VQ motif-containing genes in the *P. bretschneideri* genome was far more than in the *P. communis* genome.

We identified 32 and 8 segmentally arranged *P. bretschneideri* and *P. communis* VQ motif-containing genes, respectively, indicating that they might be the results of segmental duplication events (Figure 3 and Table S2). In *P. bretschneideri*, the high frequency of segmental duplication occurred between chromosomes 10 and 15, which possessed three segmental duplications. Interestingly, no collinearity events were arranged in tandem duplication events for both *P. bretschneideri* and *P. communis*, indicating that segmental duplication events might have played major roles in the amplification of VQ motif-containing genes in these two *Pyrus* species (Figure 3 and Table S2). 

### 3.3. Syntenic Blocks Analysis of P. bretschneideri and P. communis VQ Motif-Containing Genes

**Figure 4:**
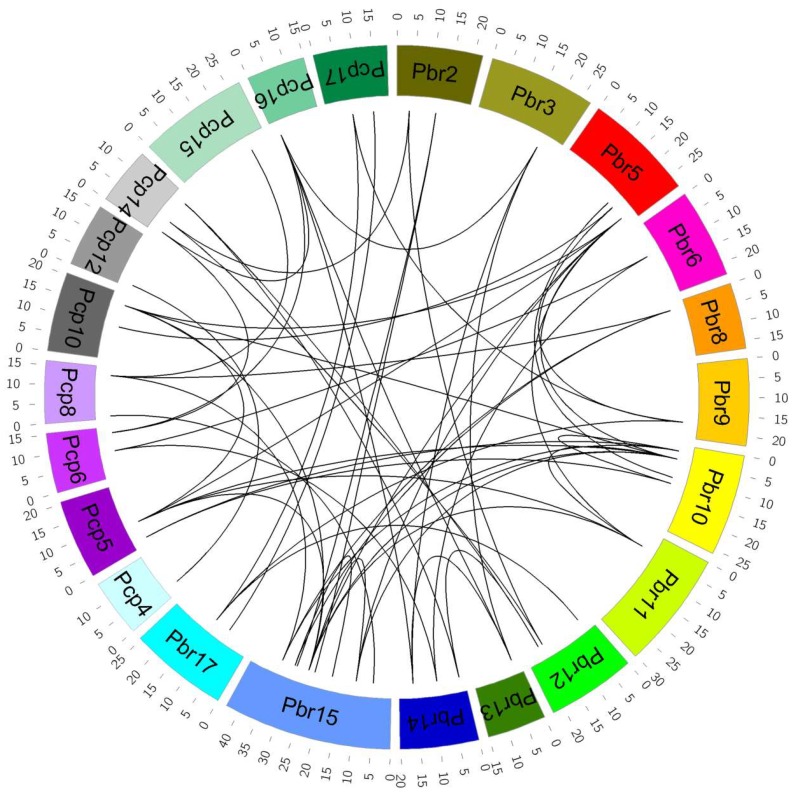
Microsynteny of VQ motif-containing genes across *P. bretschneideri* and *P. communis*. The outermost scale represents the megabases (Mb). The *P. bretschneideri* and *P. communis* chromosomes are represented by different color boxes and are labeled Pbr and Pcp, respectively. The syntenic relationships are represented by black lines. Remarkably, because *PbrVQ35*, *PbrVQ36*, *PbrVQ37*, *PbrVQ38*, *PbrVQ39*, *PbrVQ40*, *PbrVQ41*, *PcpVQ27,* and *PcpVQ28* were located on the scaffold, the related orthologous gene pairs are not shown in Figure 4.

It is well-known that synteny relations between species help us to understand the conserved biological functions and genome evolution among species. To gain insight into the evolution of *P. bretschneideri* and *P. communis* VQ motif-containing genes, the syntenic blocks were searched in the chromosomes using MCScanX software. Among them, 31 orthologous pairs were identified within these pairwise syntenic blocks (Figure 4 and Table S3). Additionally, we also observed a reciprocal homology in syntenic blocks between the *P. bretschneideri* and *P. communis* chromosomes. The syntenic blocks were distributed in most chromosomes of the *Pyrus* genomes. For *P. bretschneideri*, both chromosomes 15 and 10 presented the highest number of matches with *P. communis* VQ motif-containing genes, each of them displaying linkage with four genes in the corresponding chromosomes. The *P. communis* chromosome 5 matched the highest number of *P. bretschneideri* VQ motif-containing genes, exhibiting homology with five sequences. Curiously, the genes distributing in the same chromosome in one species matched syntenic blocks in different chromosomes from the other species, as in chromosome 15 from *P. bretschneideri* and in chromosomes 5, 8, and 10 from *P. communis* (Figure 4). 

In order to further clarify the evolutionary history of VQ motif-containing genes, we specifically analyzed the paralogous and orthologous VQ motif-containing gene pairs between *P. bretschneideri* and *P. communis*. In the present study, we identified 31 orthologous gene pairs between *P. bretschneideri* and *P. communis* (Figure 4 and Table S3). A total of 32 and 8 paralogous gene pairs were found within the *P. bretschneideri* and *P. communis* genomes, respectively. Additionally, we found that some VQ motif-containing genes were not involved in any syntenic blocks, indicating that they evolved from independent duplication events. Interestingly, we found that two or more VQ motif-containing genes from *P. communis* or *P. bretschneideri* matched one *P. bretschneideri* or *P. communis* VQ motif-containing gene, implying that these genes might have contributed to the expansion of the VQ motif-containing gene family during the course of evolution. For example, *PbrVQ7*, *PbrVQ12,* and *PbrVQ28* were orthologous to *PcpVQ5*, and *PcpVQ24* and *PcpVQ24* were orthologous to *PbrVQ34*. 

### 3.4. Gene Structural and Conserved Motifs Analysis of P. bretschneideri and P. communis VQ Motif-Containing Genes

Gene structural diversity provides the primary resources for the evolution of multi-gene families, as found in previously published articles [38,40,41]. The exon–intron distribution and organization maps of each VQ motif-containing gene were generated to understand the structural diversity of VQ motif-containing genes. As shown in Figure S1, the different numbers of exons (ranging from 2 to 13) were found among 69 VQ motif-containing genes. In *P. bretschneideri*, we found that the exon–intron number of only one homologous pair (*PbrVQ10* and *PbrVQ6*) had changed within the 32 duplicated gene pairs. In *P. communis*, the exon–intron number of two homologous pairs (*PcpVQ15* and *PcpVQ2*, *PcpVQ5* and *PcpVQ11*) had changed. For example, *PbrVQ10* and *PcpVQ2* lost four introns when compared with *PbrVQ6* and *PcpVQ15*. At the same time, we also investigated the structural diversity of orthologous genes between *P. bretschneideri* and *P. communis*. Among 31 orthologous gene pairs, we identified that the exon–intron numbers of six orthologous gene pairs (*PbrVQ6* and *PcpVQ12*, *PbrVQ7* and *PcpVQ5*, *PbrVQ12* and *PcpVQ5*, *PbrVQ28* and *PcpVQ5*, *PbrVQ16* and *PcpVQ5,* and *PbrVQ38* and *PcpVQ5*) had changed. Subsequent gene structure analysis suggested that 97.6% (40/41 genes) of *PbrVQ* genes and 78.5% (22/28 genes) of *PcpVQ* genes did not contain introns, with *PbrVQ6* (3 introns), *PcpVQ3* (12 introns), *PcpVQ5* (1 intron), *PcpVQ10* (2 introns), *PcpVQ12* (11 introns), *PcpVQ15* (8 introns), and *PcpVQ2* (4 introns) being the exceptions (Figure S1). These data were consistent with the previous studies that reported 54 VQ motif-containing genes in *Z. mays* (88.5%) [7], 37 genes in *O. sativa* (92.5%) [4], 25 genes in *P. edulis* (86.2%) [42], and 30 genes in *A. thaliana* (88.2%) [3] without introns. On the contrary, 28% (7/25) of moss VQ motif-containing genes did not possess introns. These comparative analyses (higher plants *P. bretschneideri*, *P. communis*, *P. edulis*, *A. thaliana*, *Z. mays*, and *O. sativa*, and lower plants, for example, moss) indicated that most VQ motif-containing genes had lost introns during the long evolutionary period. *PcpVQ3*, *PcpVQ12*, and *PcpVQ15*—which include 12, 11, and 8 introns, respectively—are obviously special cases, and are similar to *VQ36* in *Brassica rapa*, which contains 14 introns [11].

We also analyzed the conserved motif using the MEME software (Figure S1). In the present study, we identified twenty different motifs (Figure S1 and Table S4). All of the VQ motif-containing genes from *P. bretschneideri* and *P. communis* clearly possessed the same motif 1, which was encoded by the VQ domain. The motif analysis suggested that VQ motif-containing proteins belonging to the same subfamily possessed basically the same type of motif, which supported the phylogenetic analysis. Subsequently, we also obtained additional information from the analysis of paralogous gene pairs and orthologous gene pairs between *P. bretschneideri* and *P. communis*. Among these paralogous/orthologous gene pairs, the type of motif in each gene pair was basically consistent. The presence of the same conserved domain implied that the function of the homologous gene pairs was similar at the protein level. 

### 3.5. Expression Pattern Analysis of P. bretschneideri VQ Motif-Containing Genes by Transcriptome Data

**Figure 5:**
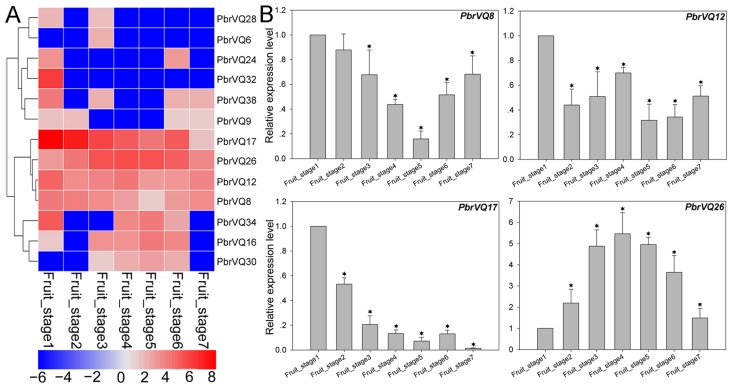
Expression profiles of *P. bretschneideri* VQ motif-containing genes during seven fruit developmental stages, including fruit_stage1 (15 days after full blooming (DAB)), fruit_stage2 (30 DAB), fruit_stage3 (55 DAB), fruit_stage4 (85 DAB), fruit_stage5 (115 DAB), fruit_stage6 (mature stage), and fruit_stage7 (fruit senescence stage). (**A**) Heat map showing expression levels of 13 *P. bretschneideri* VQ motif-containing genes in seven stages of pear fruit development. As shown in the bar at the left bottom of Figure 5, gene transcript abundance is represent by different colors in the map. The FPKM (Fragments Per Kilobase of exon model per Million mapped fragments) values of *PbVQ* genes are presented in Table S5. (**B**) Gene expression of *PbrVQ8*, *PbrVQ12*, *PbrVQ17,* and *PbrVQ26* was analyzed by real-time polymerase chain reaction (qRT-PCR). The standard error bar indicates three biological replicates. The asterisk indicates the significant difference (* *p* < 0.05) between treatment and control (fruit_stage1).

To further understand the expression patterns of VQ motif-containing genes, we analyzed publicly available RNA-seq data from the NCBI SRA (Sequence Read Archive) database for a total of eight experiments, including pollen tube development, fruit development, abiotic stress, biotic stress, and developmental biology. The sample details and treatments for these data are presented in the availability of data and materials section. In the present study, we examined the expression of *P. bretschneideri* VQ motif-containing genes in four growth stages of pollen (i.e., MP (mature pollen grain), HP (hydrated pollen grain), PT (growing pollen tube), and SPT (stopped-growth pollen tube)) (Figure S2). We found that only three *PbrVQ* genes, including *PbrVQ8*, *PbrVQ1*, and *PbrVQ23*, were expressed in one or several periods. Among them, the *PbrVQ23* gene was expressed in all periods, indicating that this gene plays an important role in pear pollen tube development. The expression patterns of *PbrVQ* genes were also investigated during pear fruit development. A total of 13/41 (31.7%) *PbrVQ* genes were relatively highly expressed in pear fruit. Among them, four genes (*PbrVQ8*, *PbrVQ12*, *PbrVQ17*, and *PbrVQ26*) were expressed in all pear fruit development stages (Figure 5 and Table S5). These genes were mainly distributed in subfamilies I, II, IX, and X. We also used the qRT-PCR experiment to confirm the expression patterns, as shown in Figure 5B. The expression trends of these genes were similar to those of RNA-seq data (Figure 5 and Table S5).

In addition, VQ motif-containing genes have been previously reported to be involved in biotic and abiotic stresses [4,6,11]. We found that the most of the *PbrVQ* genes (29 genes) presented differential expression when compared with the untreated control, while the remaining genes (12 genes) were not detectable. For example, after black spot (*Alternaria alternata*) treatment, *PbrVQ8*, *28*, *23*, *33*, *9*, *30*, *2*, *32*, *13*, *5*, *11*, *38*, *3*, *15*, *24*, *15*, *10*, *39*, and *20* were up-regulated, and *PbrVQ12*, *16*, *26*, *34*, *41*, and *6* were down-regulated (Figure S2). It is well-known that GA (Gibberellic acid) has various regulation functions in plants, such as controlling fertilization time, simulating organ growth, and early seed development. In our study, the top highly expressed gene in the *P. bretschneideri* sample by GA treatment was *PbrVQ9* (Figure S2). Our data suggested that these genes contained the potential roles in stress response.

### 3.6. Comparison of the Expression Patterns of P. bretschneideri VQ Motif-Containing Genes and Their Orthologous in P. communis during Fruit Development

**Figure 6:**
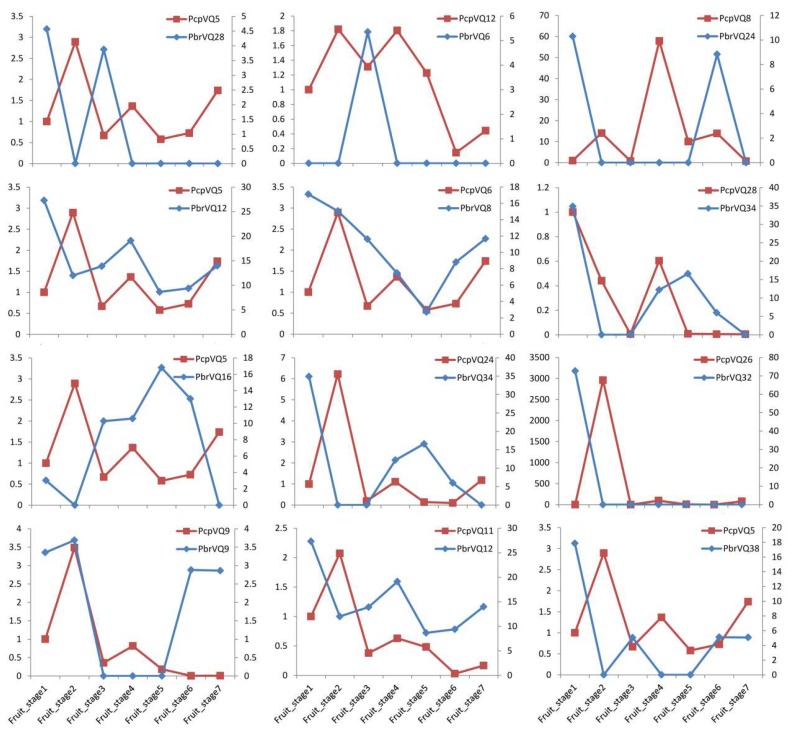
Comparison of the expression patterns of *P. bretschneideri* VQ motif-containing genes and their orthologous in *P. communis* during fruit development. X-axis represents the fruit developmental stages. Y-axis on the left indicates the relative values of FPKM in *P. bretschneideri* VQ motif-containing genes by blue line charts, and Y-axis on the right represents the relative gene expression levels of qRT-PCR in *P. communis* by red line charts. The FPKM values of *PbVQ* genes are presented in Table S5.

Pears are widely cultivated around the world, and the fruit is the focus of this study because of its economic value. As we know, the homologous gene pairs might possess similar expression patterns. In our study, in order to further understand the degree of expression diversity of VQ motif-containing genes between *P. bretschneideri* and *P. communis*, we estimated their expression correlations. In the present study, one orthologous gene pair (*PbrVQ34–PcpVQ28*) was identified to be non-divergent, two orthologous gene pairs (*PbrVQ9–PcpVQ9* and *PbrVQ8–PcpVQ6*) were ongoing divergent, and the remaining orthologous gene pairs were divergent (Figure 6 and Table S4). These results indicated that most of the orthologous VQ motif-containing gene pairs from *P. bretschneideri* and *P. communis* have undergone functional divergence (Figure 6 and Table S6). These expression patterns differ from those previously reported between *B. rapa* and *A. thaliana* orthologous VQ motif-containing gene pairs, where it was found that most of the orthologous gene pairs presented similar expression profiles under hormone and abiotic treatment [11]. The reason for this divergence might have been that the culture conditions in the orchard of the tree of both pear species were different. Our study mainly explored the divergences of orthologous gene pairs in the developmental stages of pear fruit. At the same time, gene expression profiles in both *P. bretschneideri* and *P. communis* fruits suggested that functional redundancy derived from a common ancestry for some orthologous gene pairs, and from sub-functionalization or neo-functionalization for others. 

## 4. Discussion

### 4.1. Phylogeny of P. bretschneideri and P. communis VQ Motif-Containing Gene Family

VQ motif-containing genes are involved in the regulation of various processes, such as plant growth and development, as well as the resistance to biotic and abiotic stresses. The VQ motif-containing gene family has been discovered in many species, such as *O. sativa*, *Z. mays*, *A. thaliana,* and others [3,4,5,6,7,9,11,42]. However, there is still lack of systematic analyses of VQ motif-containing genes in two *Pyrus* species (i.e., *P. bretschneideri* and *P. communis*). Subsequently, the genome-wide analyses of *P. bretschneideri* and *P. communis* VQ motif-containing genes and their regulation during pollen tube, fruit developmental processes, and/or stress responses were performed.

In the higher eukaryotes, intronless genes are very common in their genomes [43]. In our study, we found that most of the *PbrVQ* and *PcpVQ* genes are intronless, based on the gene structure analysis. Only one gene contained multiple introns in *P. bretschneideri* and five genes contained multiple introns in *P. communis*. Subsequent to the phylogenetic analysis of VQ motif-containing genes in *P. bretschneideri*, *P. communis*, *A. thaliana,* and *O. sativa*, VQ genes containing intron(s) were presented in several different subfamilies, indicating that these introns arose relatively independently and recently in *P. bretschneideri*, *P. communis*, *A. thaliana,* and *O. sativa*. The *PbrVQ* and *PcpVQ* genes identified in the present study, and those identified in other species, will facilitate further studies of plant intron evolution.

Some conserved motifs and intron–exon structures within the VQ motif-containing genes existed not only in higher plants (such as *P. bretschneideri*, *P. communis*, Arabidopsis, maize, and rice) but also in lower plants (such as moss), indicating their ancient origin in evolutionary history and their important roles in plant developmental processes. Indeed, our hypothesis was also supported by some research articles. For example, *AtVQ14* mutation produces small seeds and reduces endosperm growth, indicating that this gene might be involved in seed development [44,45]. Throughout the entire life cycle, the recessive loss-of-function *AtVQ8* mutant exhibits stunted-growth and pale-green phenotypes, indicating a predominant role in photosystem assembly or chloroplast development [3]. VQ motif-containing genes also mediate the responses to environmental stresses and pathogen infection in rice [4]; salicylic acid (SA), ethylene (ETH), powdery mildew infection, and drought in grapes [6]; low-nitrogen stress in soybeans; osmotic stresses and drought in maize [7]; and plant hormone treatments and different abiotic stresses in cabbage [11].

### 4.2. Expansion and Duplication of P. bretschneideri and P. communis VQ Motif-Containing Gene Family

The identification of duplicate genes that are retained after GWD may be challenging, as found in previously published articles [46]. Many rearrangements of the pear genome via fractionation and two GWD events can lead to loss of synteny [20,47], which causes the detection of gene pairs to be complicated. In our study, we did not distinguish between segmental duplications and GWD gene pairs, as this makes it difficult to separate these two duplication types. For pears, previous research indicated that dispersed duplication and GWD/segmental duplication were the main sources of duplicate genes [38,40,41,48]. The VQ motif-containing genes were identified in GWD/segmental duplication as a common evolutionary mechanism in *P. bretschneideri* and *P. communis*. GWD/segmental duplication events were the main driving force during the VQ motif-containing gene family expansion in pears. In the present study, 32 and 8 duplication VQ motif-containing gene pairs were identified as GWD/segmental duplication in *P. bretschneideri* and *P. communis*, respectively. However, previous researchers did not identify any duplication events in the VQ motif-containing genes of grapevine [6]. These results suggested that differences in the evolution of the VQ motif-containing gene family exist in different species. Additionally, we also explored the role of GWD events in the expansion of *P. bretschneideri* and *P. communis* VQ motif-containing gene family members. Our data suggested that the recent GWD events contributed to the expansions of *P. bretschneideri* VQ motif-containing genes.

### 4.3. Expression Analysis of P. bretschneideri VQ Motif-Containing Genes 

Previous studies reported that many VQ motif-containing genes play roles in response to biotic and abiotic stresses [3,4,6,9,11,19]. In the present study, some *PbrVQ* genes were relatively highly expressed in *P. bretschneideri* samples that were treated with GA (Gibberellic acid), salt, and black spot (*Alternaria alternata*). For example, 17 *PbrVQ* genes were induced by black spot treatments. The *PbrVQ17* was highly expressed in *P. bretschneideri* samples by GA treatment, which was consistent with *B. rapa VQ* genes in response to GA [11]. Additionally, increasingly more studies have shown that the transcription of VQ motif-containing genes is regulated by environmental and various endogenous signals, which is consistent with its different roles in plant growth and development. In *A. thaliana*, the *AtVQ14* gene is mainly associated with seed development, such that its mutation produces small seeds [44,45]. Compared with wild type plants, over-expression of *AtVQ22*, *AtVQ18*, and *AtVQ17* causes highly stunted growth of the transgenic plans [10]. The *AtVQ8* plays an important role in photosystem assembly and chloroplast development, which is consistent with its location in plastid [3]. Recently, a study has shown that the heterologous expression of *AtVQ21* in petunia and jonquil led to delayed flowering and dwarfed phenotypes in these species [49]. In our study, the expression levels of *PbrVQ* genes were detected during *P. bretschneideri* fruit development. These data indicated that the majority of the *PbrVQ* genes were differentially expressed during *P. bretschneideri* fruit development. Several *PbrVQ* genes were expressed in all periods of pear fruit development, indicating that these genes might be very important for the development of pear fruit. Previous studies have shown that AtVQ20 protein interacts with both AtWRKY2 and AtWRKY34 transcription factors to modulate pollen development and function [50]. Therefore, we detected the expression patterns of the *PbrVQ* genes during pear pollen development. The *PbrVQ23*, the homolog of *AtVQ20*, was highly expressed during pear pollen development when compared with its level in other examined tissues. Taken together, our study suggested that the VQ motif-containing genes were extensively involved in pollen tube and fruit growth and development.

## 5. Conclusions

In our study, a systematic study was performed on the genome-wide identification, phylogenetic relationship, characterization, and expression analysis of VQ domain-containing genes in both *P. bretschneideri* and *P. communis*. By combining a phylogenomics approach at the genome level with expression pattern analysis during fruit development, we have increased functional divergences and expansion patterns in the VQ motif-containing genes of *P. bretschneideri* and *P. communis* that evolved from the same common ancestry. Finally, this study illustrates the decisive contribution of GWD/segmental duplication events to the evolution of genes, and as a foundation for future exploration of the functional divergences of the VQ domain-containing genes in both *P. bretschneideri* and *P. communis*. 

## Supplementary Materials

The following are available online at http://www.mdpi.com/2073-4425/9/4/224/s1, Data S1: Scripts used in Section 2.2, Section 2.3 and Section 2.4. Figure S1: Exon–intron structure and conserved motifs analysis of *P. bretschneideri* and *P. communis* VQ motif-containing genes. Figure S2: The heatmap of the expression of *P. bretschneideri* VQ motif-containing genes in different tissues or pollen tube development. *PbrVQ* gene expression levels during pollen germination and pollen tube growth, including MP (Mature pollen grains), HP (Hydrated pollen grains), PT (Pollen tube), SPT (Stop-growth pollen tube), and *PbrVQ* gene expression levels in different conditions, including pear leaf with inoculated distilled water (Leaf_CK), pear leaf with inoculated black spot (*Alternarlia alternate*) 2 (Leaf_T2), pear leaf with inoculated black spot (*Alternarlia alternate*) 3 (Leaf_T3), pear fruit with no any treatment (Fruit_CK), pear fruit with Gibberellin treatment (Fruit_GA), pear leaf with salt treatment (Leaf_Salt), pear fruit pericarp-russet (pericarp-russet), and pear fruit pericarp-green (pericarp-green). Table S1: Primers in this study. Table S2: Ka/Ks analysis of between paralogous VQ motif-containing gene pairs in *Pyrus*. Table S3: Homologous analyses of VQ motif-containing genes between *P. bretschneideri* and *P. communis*. Table S4: All MEME motif sequences in both *P. bretschneideri* and *P. communis* VQ motif-containing proteins. Table S5: The FPKM values of *PbVQ* genes in different tissues or treatments. Table S6: Divergence analysis of orthologous VQ motif-containing gene pairs among *P. bretschneideri* and *P. communis.*
