# Supplementary material for: Genome Wide Identification, Evolutionary, and Expression Analysis of VQ Genes from Two Pyrus Species
Source: Genes (Basel). 2018 Apr 23;9(4):224. doi: 10.3390/genes9040224 (PMC5924566; doi:10.3390/genes9040224)
Supplement: Supplementary file 1 [file genes-09-00224-s001.zip › Supplementary Data 1. Scripts.docx]

**Section 2.2**

perl ..\bin\circos -conf ..\tutorials\5\1\circos.conf

The circos.conf file is as follows:

<<include colors_fonts_patterns.conf>>

<<include ideogram.conf>>

<<include ticks.conf>>

<image>

<<include etc/image.conf>>

</image>

karyotype = data/karyotype/1.txt

chromosomes_units = 1000000

chromosomes = hs1;hs2;hs3;hs4;hs5;hs6;hs7;hs8;hs9;hs10;hs11;hs12;hs13;hs14;hs15;hs16;hs17;

chromosomes_display_default = no

# chromosomes_radius = hs2:0.9r;hs3:0.8r

# Links (bezier curves or straight lines) are defined in <links> blocks.

# Each link data set is defined within a named <link> block. The name

# of the block is arbitrary, but must be unique.

#

# As with highlights, parameters defined

# in the root of <links> affect all data sets and are considered

# global settings. Individual parameters value can be refined by

# values defined within <link> blocks, or additionally on each

# data line within the input file.

<links>

z = 0

radius = 0.975r

bezier_radius = 0.2r

<link>

bezier_radius = undef

show = yes

color = dgrey

thickness = 5

file = data/5/3.txt

record_limit = 5000

</link>

</links>

<<include etc/housekeeping.conf>>

#track_defaults* = undef

**Section 2.3**

#!usr/bin/perl

my $header;

my $seq = "";

my $fastaFile = "Your fasta file";

my %seq;

open IN, "$fastaFile" or die "Cannot find the specified fasta file $fastaFile";

while(my $line=<IN>){

chomp($line);

if ($line=~/^>/){ #the header line

if (length $seq > 0){ #not the first line

$seq{$header} = $seq;

}

$header = substr($line,1); #remove >

$seq = "";

}else{

$seq.=$line;

}

}

$seq{$header} = $seq;

close IN;

open IN, "your positiion file";

open OUT, ">result file";

while (my $line = <IN>){

chomp ($line);

my ($chr,$pos)=split (":",$line);

my ($start,$end)=split ("-",$pos);

my $seq = $seq{$chr};

my $part = substr($seq,$start, $end-$start); #add +1 or -1 after testing

print OUT ">$line\n$part\n";

}

close IN;

close OUT;

**Section 2.4**

data=read.table("table.txt",sep="\t",header=T)

library(pheatmap)

head(data)

id=data[,1]

data=data[,-1]

data=log2(data+0.01)

colnames(data)=c("1h","3h","6h","12h","24h","1h","3h","6h","12h","24h","1h","3h","6h","12h","1h","3h","6h","12h","24h")

head(id)

id=as.vector(id)

rownames(data)=id

head(data)

data=log2(data+0.01)

pheatmap(data,fontsize=14, fontsize_row=5,fontsize_col=10,cluster_row=TRUE,

cluster_col=F,legend = TRUE,border_color="white",

show_rownames=T,show_colnames=T,

color = colorRampPalette(rev(c("red","gray90","blue")))(102),

display_numbers=F

)

dev.off()
