# Supplementary figures and images for: Genome Wide Identification, Evolutionary, and Expression Analysis of VQ Genes from Two Pyrus Species
Source: Genes (Basel). 2018 Apr 23;9(4):224. doi: 10.3390/genes9040224 (PMC5924566; doi:10.3390/genes9040224)

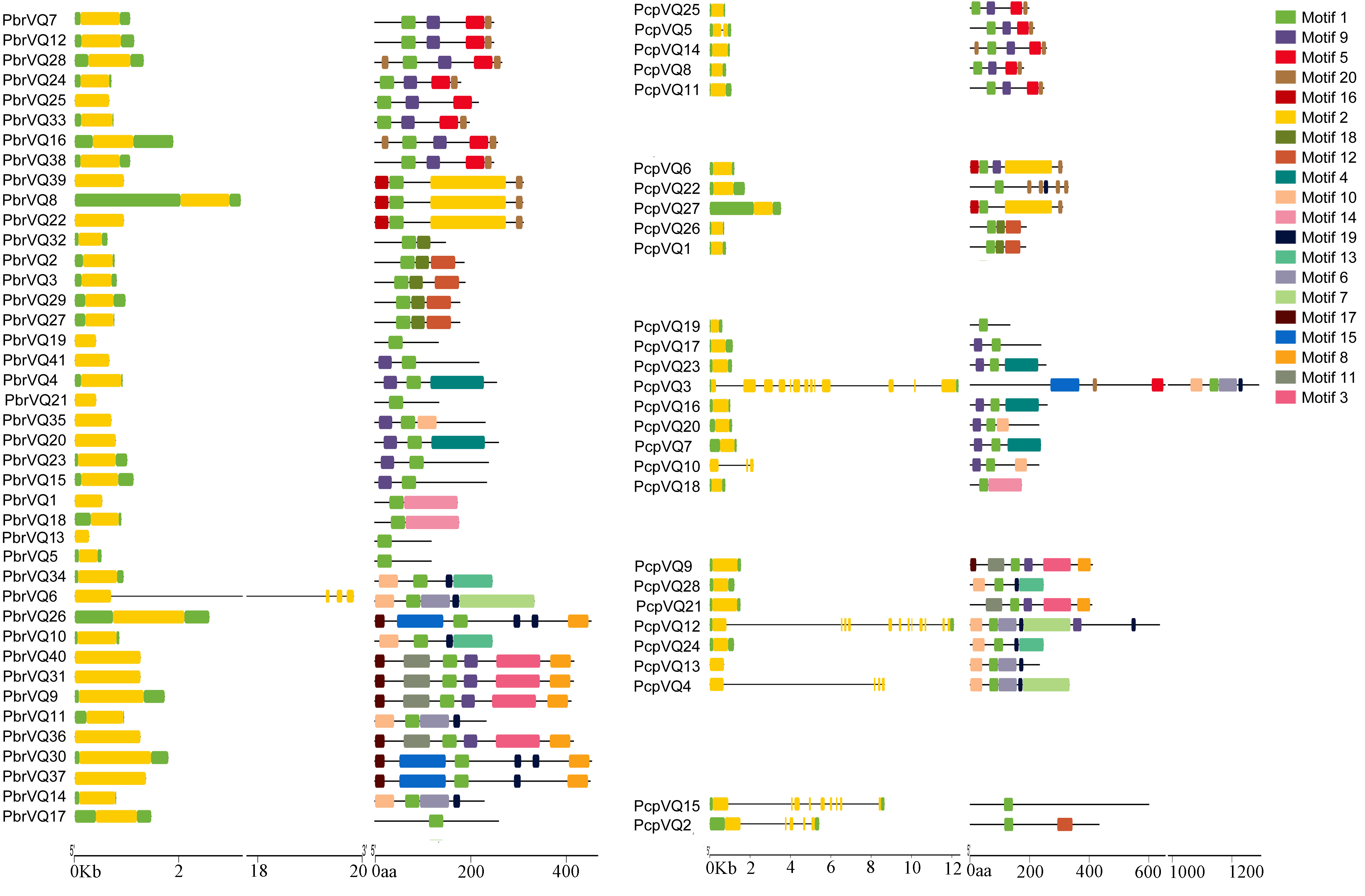

Supplement: Supplementary file 1 [file genes-09-00224-s001.zip › Figure S1.tif]

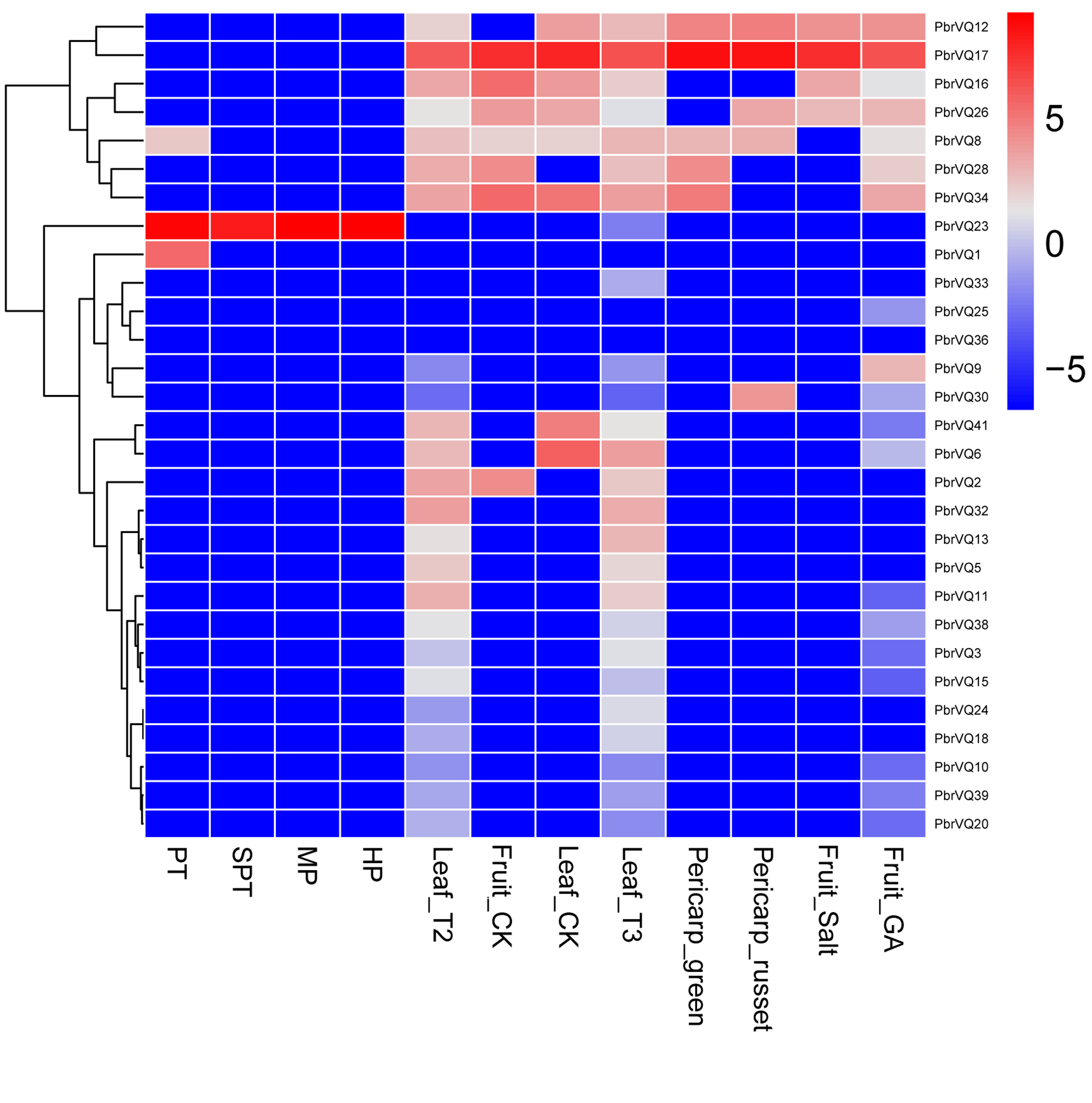

Supplement: Supplementary file 1 [file genes-09-00224-s001.zip › Figure S2.tif]
